# Role of remnant cholesterol in the relationship between physical activity and diabetes mellitus: an intermediary analysis

**DOI:** 10.3389/fpubh.2024.1322244

**Published:** 2024-03-12

**Authors:** Zihua Yang, Hao Chen, Fengxia Lai, Jingjing Zhang, Shihong Wang, Shuang Wang, Yongze Chen, Zhenhua Mai, Ling Luo, Danli Kong, Yuanlin Ding

**Affiliations:** ^1^Department of Epidemiology and Medical Statistics, School of Public Health, Guangdong Medical University, Dongguan, Guangdong, China; ^2^Affiliated Hospital of Guangdong Medical University, Zhanjiang, Guangdong, China; ^3^School of Public Health and Emergency Management, South University of Science and Technology of China, Shenzhen, Guangdong, China

**Keywords:** diabetes mellitus, physical activity, remnant cholesterol, intermediary analysis, National Health and Nutrition Examination Survey

## Abstract

**Objective:**

The purpose of this investigation was to evaluate the potential link between physical activity (PA) and the heightened susceptibility to diabetes mellitus (DM), by examining whether remnant cholesterol (RC) might act as a mediator in this correlation.

**Methods:**

The research utilized data from the National Health and Nutrition Examination Survey, spanning from 2005 to 2018. Various statistical analyses were conducted for continuous and categorical variables, including the *t*-test, ANOVA, and χ^2^ test. Logistic regression was employed to analyze the association between PA and DM across three distinct models. Mediation analysis was also conducted to assess the potential mediation effects of RC.

**Results:**

The study encompassed a total of 9,149 participants, and it was observed that individuals with DM exhibited lower levels of PA. Furthermore, PA levels were found to be associated with all participant characteristics except poverty income ratio, fasting blood glucose, and HOMA-IR (*p* < 0.05). After adjusting for covariates (Model 3), individuals with high PA levels demonstrated a decreased likelihood of developing DM compared to those in the low PA group (*OR*: 0.73, 95%*CI*: 0.54–0.99). A significant dose–response relationship was identified (*p* < 0.05). No interaction between PA and RC in relation to DM risk was detected, and RC was found to serve as a mediator in the connection between PA and DM. After considering covariates, the mediating effect of RC between PA and DM weakens.

**Discussion:**

Our findings suggest that higher levels of PA are linked to a reduced risk of DM in U.S. adults, with RC likely playing a mediating role.

## Introduction

The global prevalence of diabetes mellitus (DM) is escalating, impacting more than 537 million adults aged 20–79 worldwide (1, 2) and emerging as a significant threat to global public health. Various factors, including gender, age, race, and obesity, contribute to the risk of DM (3, 4). In the context of evolving lifestyles, the role of physical activity (PA) as a crucial determinant of health has garnered substantial attention (5–7). Numerous studies indicate that elevated PA levels are associated with reduced glycosylated hemoglobin (HbA1c) levels and a lower incidence of DM (8–10). Moreover, leisure-time PA has demonstrated efficacy in mitigating the risk of DM-related mortality (11). These findings underscore the pivotal role of PA in diminishing the risk of DM.

Remnant cholesterol (RC) refers to the cholesterol content found in triglyceride-enriched lipoproteins, which include extremely low-density lipoprotein (VLDL), low-density lipoprotein (LDL), and chylomicron residues (12, 13). Research has indicated that RC particles are not only richer than LDL-C particles but also larger and carry a greater cholesterol load, suggesting that RC may pose a potential threat to pancreatic β cells (14). It is noteworthy that an increase in RC levels not only elevates the risk of major vascular diseases and complications related to DM but also heightens the risk of developing full-blown DM in individuals with prediabetes and high RC levels (15–19). Moreover, Hu et al. confirmed that RC was superior to LDL in the association with DM (20). Recent meta-analyses underscore the beneficial effects of higher levels of exercise on various health markers, including fasting blood glucose, total cholesterol, and triglycerides, suggesting the potential role of PA in regulating blood lipid levels (12, 13, 21). Additionally, as a component of the exercise regimen, resistance training has been associated with a decrease in RC levels, highlighting the connection between specific types of PA and lipid metabolism (22). Building upon the relationship between PA, RC, and DM mentioned above, we speculate that RC may play a crucial role in the complex interplay between PA and DM.

In this context, our study utilized data from the National Health and Nutrition Examination Survey (NHANES) to comprehensively examine the interrelationships among PA, RC, and DM. By investigating the potential mediating role of RC, we aim to offer valuable insights, furnish information for more effective interventions, and ultimately contribute to global efforts aimed at reducing the burden of DM.

## Methods

### Participants and study design

The current study conducted a retrospective cross-sectional analysis utilizing data from the continuous NHANES, spanning the years 2005 to 2018. NHANES is a comprehensive and multi-stage survey program, administered by the National Center for Health Statistics (NCHS) (23), with the primary objective of evaluating the health and nutritional status of the noninstitutionalized population in the United States. Commencing in 1999, continuous NHANES has consistently collected a wide array of information encompassing demographics, socioeconomic factors, dietary habits, and health-related data from selected participants, following a biennial cycle. The participation of individuals was contingent on obtaining informed consent, and the research protocol adhered to for conducting the NHANES survey had received approval from the NCHS Research Ethics Review Board. Comprehensive insights into the survey’s design and response rate can be accessed on the NHANES official website^1^ (24).

**Figure 1:**
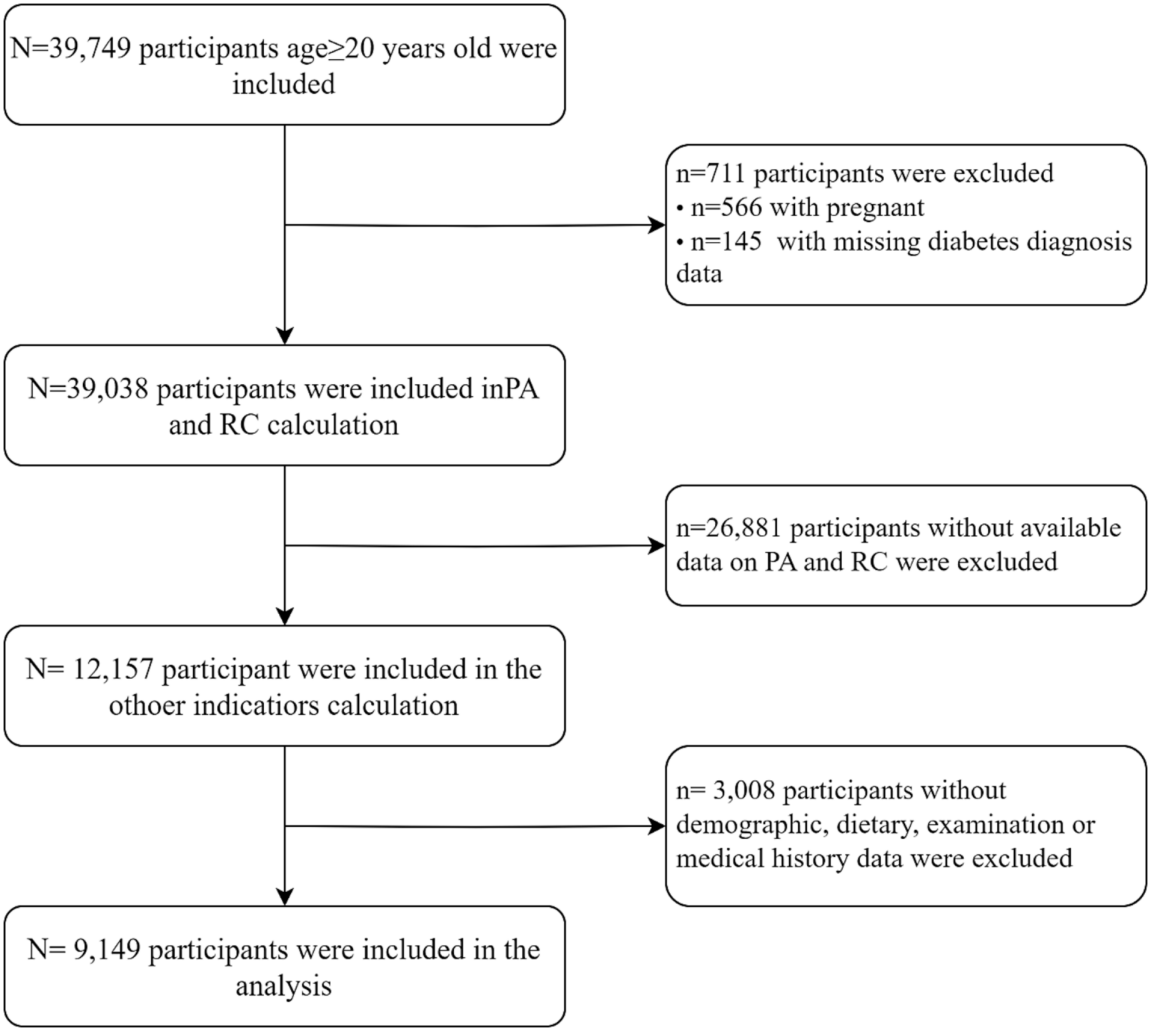
Flow chart of the study design.

Among the 39,749 adult participants (aged≥20 years) who were included, we excluded the following: (1) pregnant participants (*n* = 39,183); (2) individuals lacking data regarding a diabetes diagnosis (*n* = 39,038); (3) those without available data on physical activity and remnant cholesterol (*n* = 12,157); (4) those without demographic data [gender, age, race, education level, marital status, health insurance, sleepiness level, alcohol consumption, smoking habits, poverty index ratio (PIR)] or dietary data [dietary inflammation index (DII)] or examination data [body mass index (BMI), glycosylated hemoglobin (HbA1c), fasting blood glucose (FBG), fasting serum insulin (FINS)] or medical history data [hypertension, cardiovascular disease (CVD)] (*n* = 9,149) (Figure 1).

### Physical activity

Each participant completed a thorough physical activity questionnaire, which covered their physical activities during two different time frames: the past 30 days (from 2005 to 2006) and 1 week (from 2007 to 2018). This questionnaire collected detailed information about the activity type, frequency, intensity, and duration of physical activities conducted. Metabolic equivalent (MET) scores were calculated for specific activities based on activity type and intensity (25). These MET scores were then multiplied by the average duration and frequency of participation within the past 30 days or week. This multiplication resulted in the determination of MET minutes per 30 days (MET min/30d) or MET minutes per week (MET min/week) for each activity. Then we aggregated the MET minutes per 30 days or week for all reported activities. Subsequently, for participants who reported their physical activity monthly, we divided the total MET minutes per 30 days by 30 and then multiplied this figure by 7 to compute the total MET minutes per week for each participant. Afterward, participants were classified into three groups based on the standard scoring criteria of the International Physical Activity Questionnaire (IPAQ): low (<600 MET-min/week), moderate (600–3,000 MET-min/week), and high (≥3,000 MET-min/week) (26).

### Diabetes mellitus

To define whether a participant had diabetes, this study utilized a combination of questionnaire data and examination data, and diabetes was judged to be present if any of the following criteria were met: (1) doctor told you have diabetes; (2) HbA1c (%) > 6.5; (3) fasting glucose (mmol/l) ≥ 7.0; (4) random blood glucose (mmol/l) ≥ 11.1; (5) two-hour OGTT blood glucose (mmol/l) ≥ 11.1; (6) Use of diabetes medication or insulin.

### Remnant cholesterol

RC (mmol/L) was calculated from a standard lipid profile of the patient in a fasting state as total cholesterol (TC) (mmol/L) minus LDL-C (mmol/L) minus HDL-C (mmol/L).

### Study covariates

The included covariates included gender (female or male), education level (less than high school, completed high school and more than high school), race (non-Hispanic white, non-Hispanic black, Mexican American, and other), marital status (never married, married, and separated, divorced or widowed), age, PIR, health insurance (yes or no), cardiovascular disease (CVD), hypertension, body mass index (BMI), dietary inflammation index (DII), sleepiness level, alcohol use (former, never and now), smoking status (former, never and now), glycosylated hemoglobin (HbA1c), fasting blood glucose (FBG), HOMA-IR.

### Statistical analysis

The recommended weighting method was used to analyze NHANES data. The baseline characteristics of the study population were reported as the mean (standard error, SEM) of continuous variables and the number (percentage) of categorical variables. We used the logistic regression model to estimate the *OR* and 95% *CI*.

For our main analysis, PA was included as a continuous variable, and due to its non-normal distribution, we opted for a logarithmic transformation to better suit our statistical analyses. We reported an estimate of the effect of 1-SD increase (z-score) in PA.

This method assumes a linear relationship between PA and DM. To check the hypothesis, we then used a multivariate restriction cubic spline and placed four nodes at the 5th, 35th, 65th, and 95th percentiles of the PA distribution nodes to provide a graphical representation. The spline curve allowed us to test whether there was a significant difference with the linear correlation. Finally, we categorized PA into three groups based on IPAQ criteria and considered the low group as the reference category in the logistic model.

Three models were proposed: the first model did not adjust for confounding factors; the second model was further adjusted for known risk factors for DM and potential confounding factors, such as age, gender, race, education level, marital status, smoking status, alcohol use, sleepiness level, health insurance status, PIR, BMI, CVD, DII, HbA1c, FBG, and HOMA-IR; and the third model further adjusted RC.

The interaction of PA and RC on the risk of DM was estimated by including a multiplication term between the two variables in the logistic model. Considering the statistical significance, we examined the association between PA and DM stratified by RC category. Regression-based mediation analysis was used to distinguish the direct effect of adherence to PA on the risk of DM and the indirect effect mediated by RC. Three estimates were obtained as follows:

- Total effect, that is, the overall association between PA and the risk of DM, including the association mediated by RC;
- Direct effect, that is, the association between PA and DM risk, adjusted according to RC;
- Indirect effect, that is, the association between PA and DM risk mediated by RC.

In addition, to effectively understand the complex relationship between PA, RC, and DM, we used a counterfactual mediation model that allows exposure-medium interaction. All statistical analyses were performed using the software package R (The R Foundation).^2^ A two-tailed *p*-value of <0.05 was considered statistically significant.

## Results

### Characteristics of participants

In this study, a grand total of 9,149 individuals were enlisted, out of which 1,566 participants were incorporated into the DM cohort. Compared with nondiabetics, participants with DM had higher rates of being non-Hispanic black, being divorced, widowed or separated, having less than a high school education, having previously consumed alcohol, having previously smoked cigarettes, having health insurance, and having hypertension and CVD. Participants with DM were also older and had higher HbA1c, FBG, BMI, DII, HOMA-IR, and RC. In addition, diabetic participants were less PA than non-diabetic participants. The general characteristics of the study population are shown in Table 1.

**Table 1:** Weighted characteristics of the study population*.

Characteristics	Total (*n* = 9,149)	DM		χ^2^/*t*	*P*-value
		No (*n* = 7,583)	Yes (*n* = 1,566)		
Age	45.65 ± 0.30	43.96 ± 0.32	57.45 ± 0.45	25.713	< 0.001
PIR	3.15 ± 0.04	3.17 ± 0.04	3.00 ± 0.06	−3.120	0.002
HbA1c	5.53 ± 0.01	5.34 ± 0.01	6.87 ± 0.05	29.074	< 0.001
FBG	5.77 ± 0.02	5.45 ± 0.01	8.07 ± 0.10	27.348	< 0.001
BMI	28.62 ± 0.11	28.03 ± 0.11	32.76 ± 0.25	17.620	< 0.001
DII	1.31 ± 0.04	1.29 ± 0.04	1.48 ± 0.08	2.553	0.012
HOMA-IR	3.19 ± 0.06	2.64 ± 0.04	6.97 ± 0.30	14.232	< 0.001
RC	0.59 ± 0.01	0.57 ± 0.01	0.74 ± 0.02	11.944	< 0.001
PA	4337.21 ± 103.00	4494.65 ± 110.31	3236.64 ± 157.62	−7.444	< 0.001
Ln PA	7.46 ± 0.03	7.49 ± 0.03	7.18 ± 0.04	−6.970	< 0.001
Gender				0.346	0.558
Male	4,825(51.90)	3,949(51.76)	876(52.91)		
Female	4,324(48.10)	3,634(48.24)	690(47.09)		
Race				9.558	< 0.001
Mexican American	1,327(7.30)	1,055(7.08)	272(8.80)		
Non-Hispanic white	4,281(71.10)	3,662(71.81)	619(66.18)		
Non-Hispanic black	1751(9.98)	1,383(9.51)	368(13.30)		
Other Hispanic and other	1790(11.61)	1,483(11.60)	307(11.72)		
Marital status				44.532	< 0.001
Married or living with partner	5,642(65.06)	4,646(64.77)	996(67.10)		
Widowed, divorced, or separated	1752(15.94)	1,333(14.84)	419(23.64)		
Never married	1755(19.00)	1,604(20.39)	151(9.26)		
Education level				19.512	< 0.001
Elementary and secondary education	1769(12.82)	1,363(12.25)	406(16.81)		
High school	2075(22.34)	1,670(21.51)	405(28.14)		
Bachelor degree or higher	5,305(64.84)	4,550(66.24)	755(55.05)		
Alcohol use				37.213	< 0.001
Never	1,045(9.05)	814(8.44)	231(13.30)		
Former	1,311(11.56)	965(10.61)	346(18.19)		
Now	6,793(79.39)	5,804(80.95)	989(68.51)		
Smoking status				18.573	< 0.001
Never	5,038(55.00)	4,245(55.58)	793(50.96)		
Former	2,279(25.34)	1760(24.11)	519(33.95)		
Now	1832(19.65)	1,578(20.31)	254(15.09)		
Hypertension				359.45	< 0.001
No	5,612(65.71)	5,112(70.40)	500(32.97)		
Yes	3,537(34.29)	2,471(29.60)	1,066(67.03)		
CVD				190.56	< 0.001
No	8,331(92.75)	7,082(94.45)	1,249(80.86)		
Yes	818(7.25)	501(5.55)	317(19.14)		
Sleepiness level				4.087	0.019
Short sleep	3,038(29.42)	2,476(28.92)	562(32.97)		
Normal	5,231(61.56)	4,398(62.22)	833(56.95)		
Long sleep	880(9.02)	709(8.87)	171(10.08)		
Health insurance				25.574	< 0.001
No	1984(17.07)	1753(17.86)	231(11.55)		
Yes	7,165(82.93)	5,830(82.14)	1,335(88.45)		
Two groups of RC				160.92	< 0.001
Low	4,579(50.39)	4,040(53.12)	539(31.33)		
High	4,570(49.61)	3,543(46.88)	1,027(68.67)		
Three groups of PA				16.036	< 0.001
Low	2,135(22.49)	1,699(21.78)	436(24.27)		
Moderate	3,623 (40.62)	2,954(40.10)	669(44.23)		
High	3,391(36.89)	2,930(38.12)	461(28.29)		

*Rate and mean ± standard deviation were weighted; *t*-test was used for continuous variable and χ^2^-test was used for categorical variables. PIR, poverty income ratio; HbA1c, glycosylated hemoglobin; FBG, fasting blood glucose; BMI, body mass index; DII, dietary Inflammatory Index; HOMA-IR, homeostatic model assessment for insulin resistance; RC, remnant cholesterol; PA, physical activity; Ln, natural logarithm; CVD, cardiovascular disease; DM, diabetes mellitus.

### Characterization of participants according to PA

Characteristics of participants divided after grouping PA according to IPAQ criteria are shown in Table 2. Higher levels of PA were associated with lower age, HbA1c, RC, and DII. Meanwhile, compared with the group with low levels of PA, the intermediate and high-level groups had higher proportions of males, unmarried, current alcohol drinkers, and former smokers, and conversely, lower proportions of RC levels, suffering from hypertension, CVD, and DM.

**Table 2:** Characteristics of adults aged 20 and above grouped by PA*.

Characteristics	Total (*n* = 9,149)	Three groups of PA			χ^2^/*t*	*P*-value
		Low (*n* = 2,135)	Moderate (*n* = 3,623)	High (*n* = 3,391)		
Age	45.65 ± 0.30	48.78 ± 0.55	46.82 ± 0.41	42.47 ± 0.37	−7.305	< 0.001
PIR	3.15 ± 0.04	3.22 ± 0.06	3.32 ± 0.05	2.93 ± 0.04	−1.585	0.116
HbA1c	5.53 ± 0.01	5.57 ± 0.02	5.53 ± 0.02	5.52 ± 0.02	−2.278	0.025
FBG	5.77 ± 0.02	5.81 ± 0.04	5.77 ± 0.03	5.76 ± 0.03	−0.943	0.348
BMI	28.62 ± 0.11	29.09 ± 0.20	28.48 ± 0.15	28.49 ± 0.16	−3.033	0.003
DII	1.31 ± 0.04	1.52 ± 0.06	1.25 ± 0.05	1.26 ± 0.04	−3.816	< 0.001
HOMA-IR	3.19 ± 0.06	3.37 ± 0.11	3.21 ± 0.08	3.05 ± 0.08	−1.818	0.071
RC	0.59 ± 0.01	0.63 ± 0.01	0.59 ± 0.01	0.57 ± 0.01	−4.931	< 0.001
Gender					83.189	< 0.001
Male	4,825(51.90)	939(41.87)	1756(47.54)	2,130(62.82)		
Female	4,324(48.10)	1,196(58.13)	1867(52.46)	1,261(37.18)		
Race					8.251	< 0.001
Mexican American	1,327(7.30)	320(6.80)	433(5.68)	574(9.38)		
Non-Hispanic white	4,281(71.10)	988(71.18)	1759(73.14)	1,534(68.82)		
Non-Hispanic black	1751(9.98)	446(10.85)	641(8.65)	664(10.93)		
Other Hispanic and other	1790(11.61)	381(11.17)	790(12.54)	619(10.87)		
Marital status					11.888	< 0.001
Married or living with partner	5,642(65.06)	1,315(66.91)	2,280(66.15)	2047(62.73)		
Widowed, Divorced, or Separated	1752(15.94)	504(19.15)	672(15.71)	576(14.25)		
Never married	1755(19.00)	316(13.94)	671(18.14)	768(23.03)		
Education level					20.067	< 0.001
Elementary and secondary education	1769(12.82)	442(12.71)	564(10.08)	763(15.92)		
High school	2075(22.34)	514(23.89)	724(18.78)	837(25.31)		
Bachelor degree or higher	5,305(64.84)	1,179(63.40)	2,335(71.14)	1791(58.77)		
Alcohol use					6.639	< 0.001
Never	1,045(9.05)	295(10.96)	421(9.11)	329(7.83)		
Former	1,311(11.56)	381(14.63)	497(10.77)	433(10.55)		
Now	6,793(79.39)	1,459(74.41)	2,705(80.12)	2,629(81.62)		
Smoking status					8.424	< 0.001
Never	5,038(55.00)	1,208(57.66)	2072(56.82)	1758(51.37)		
Former	2,279(25.34)	540(24.17)	928(26.31)	811(24.99)		
Now	1832(19.65)	387(18.17)	623(16.86)	822(23.63)		
Hypertension					12.526	< 0.001
No	5,612(65.71)	1,175(60.26)	2,211(66.01)	2,226(68.72)		
Yes	3,537(34.29)	960(39.74)	1,412(33.99)	1,165(31.28)		
CVD					3.609	0.030
No	8,331(92.75)	1919(91.95)	3,269(92.16)	3,143(93.88)		
Yes	818(7.25)	216(8.05)	354(7.84)	248(6.12)		
Sleepiness level					3.802	0.005
Short sleep	3,038(29.42)	746(30.41)	1,109(26.83)	1,183(31.67)		
Normal	5,231(61.56)	1,174(59.76)	2,164(64.43)	1893(59.50)		
Long sleep	880(9.02)	215(9.83)	350(8.74)	315(8.83)		
DM					16.036	< 0.001
No	7,583(82.88)	1,699(84.71)	2,954(86.37)	2,930(90.40)		
Yes	1,566(17.12)	436(15.29)	669(13.63)	461(9.60)		
Health insurance					26.560	< 0.001
No	1984(17.07)	415(16.01)	634(13.53)	935(21.62)		
Yes	7,165(82.93)	1720(83.99)	2,989(86.47)	2,456(78.38)		
Two groups of RC					11.932	< 0.001
Low	4,579(50.39)	985(45.75)	1808(49.72)	1786(53.96)		
High	4,570(49.61)	1,150(54.25)	1815(50.28)	1,605(46.04)		

*Rate and mean ± standard deviation were weighted; *t*-test was used for continuous variable and χ^2^-test was used for categorical variables. PIR, poverty income ratio; HbA1c, glycosylated hemoglobin; FBG, fasting blood glucose; BMI, body mass index; DII, dietary Inflammatory Index; HOMA-IR, homeostatic model assessment for insulin resistance; RC, remnant cholesterol; PA, physical activity; CVD, cardiovascular disease; DM, diabetes mellitus.

### Association between PA and DM

**Figure 2:**
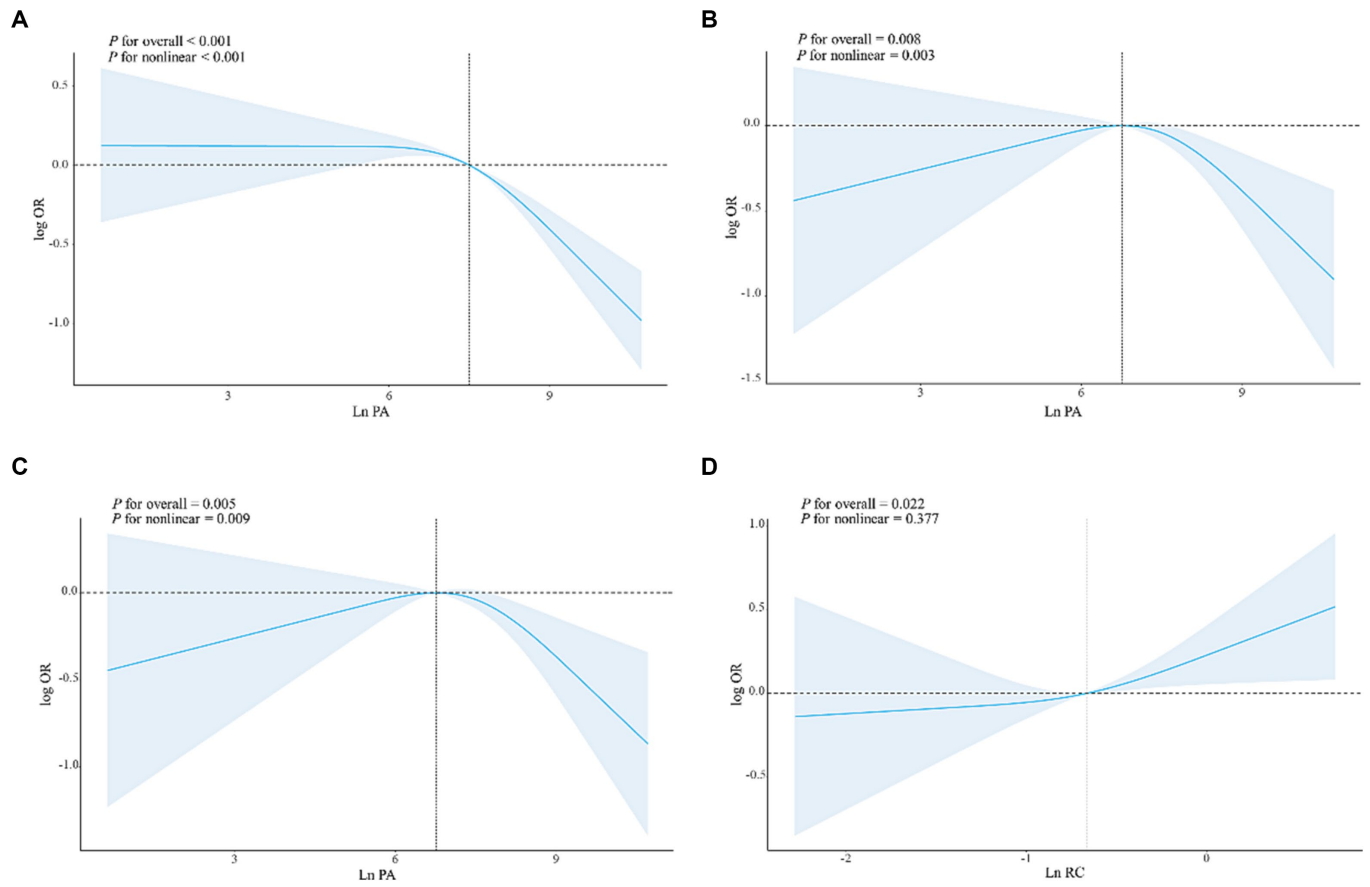
The dose–response relationships of PA/RC with DM in all participants. Results were from restricted cubic spline models. **(A)** The dose–response relationship between PA and DM without adjusting for any covariates. **(B)** The dose–response relationship between PA and DM adjusted for age, PIR, FBG, BMI, DII, HOMA-IR, gender, race, marital status, education level, alcohol use, smoking status, hypertension, CVD, sleepiness level, and health insurance. **(C)** The dose–response relationship between PA and DM adjusted for age, PIR, FBG, BMI, DII, HOMA-IR, gender, race, marital status, education level, alcohol use; smoking status, hypertension, CVD, sleepiness level and health insurance, and RC. **(D)** The dose–response relationship between RC and DM adjusted for age, PIR, FBG, BMI, DII, HOMA-IR, gender, race, marital status, education level, alcohol use; smoking status, hypertension, CVD, sleepiness level and health insurance, and PA.

The findings from Table 3 indicate that there is an inverse relationship between PA and the risk of developing DM in the high-level group compared to the low-level group in Model 1 [*OR*: 0.59 (95% *CI*: 0.49–0.71)]. This negative association between high PA levels and DM risk is also observed in Model 2 and Model 3 [*OR*: 0.75 (95% *CI*: 0.58–0.98), *OR*: 0.76 (95% *CI*: 0.58–0.99), respectively]. Furthermore, a significant dose–response relationship is evident in all three models (*P*-trend <0.05). Similar results are obtained when analyzing a 1-SD increase in PA in Model 1 [*OR*: 0.82 (95% *CI*: 0.77–0.87)], Model 2 [*OR*: 0.82 (95% *CI*: 0.72–0.95)], and Model 3 [*OR*: 0.83 (95% *CI*: 0.72–0.96)]. In addition, we observed a positive correlation between high RC levels and DM risk in Model 1, Model 2, and Model 3 [*OR*: 2.48 (95% *CI*, 2.15–2.87), *OR*: 2.34 (95% *CI*: 1.99–2.75), *OR*: 1.29 (95% *CI*, 1.02–1.62)], and significant dose–response relationships (P trend < 0.05) were observed (Supplementary Table S1). In the results of the restricted cubic spline analysis in Figure 2, there is a non-linear relationship between PA and DM risk in the three models (*P*_non-linear_ < 0.05), indicating that after the inflection point, the DM risk gradually decreases with the increase of PA level (Figures 2A–C). In the restricted cubic spline analysis of RC and DM, there is a linear relationship in Model 3 (Figure 2D) (*P*_non-linear_ > 0.05). As RC increases, the risk of DM also gradually increases, and the same results were observed in Model 1 and Model 2 (Supplementary Figure S1).

**Table 3:** DM risk based on PA grouping*.

Three groups of PA	Model 1 *OR* (95% *CI*)	Model 2 *OR* (95% *CI*)	Model 3 *OR* (95% *CI*)
Low	Reference		
Moderate	0.87(0.72–1.06)	1.09(0.88–1.35)	1.09(0.88–1.36)
High	0.59(0.49–0.71)	0.75(0.58–0.98)	0.76(0.58–1.00)
*P*-trend	<0.001	0.032	0.042
For 1-SD increase	0.82(0.77–0.87)	0.821(0.72–0.95)	0.83(0.72–0.96)

*All estimates were weighted. PA, physical activity; DM, diabetes mellitus; OR, odds ratio; CI, confidence interval. Model 1: Did not adjust any covariates; Model 2: Adjusted for age, PIR, FBG, BMI, DII, HOMA-IR, gender, race, marital status, education level, alcohol use; smoking status, hypertension, CVD, sleepiness level and health insurance; Model 3: Adjusted for age, PIR, FBG, BMI, DII, HOMA-IR, gender, race, marital status, education level, alcohol use; smoking status, hypertension, CVD, sleepiness level and health insurance, and RC; For 1-SD increase: Using PA with natural logarithms.

### PA and DM risk stratified by RC category

According to Table 4, the occurrence of PA and DM was primarily observed in the high level of PA in Model 1, with a risk ratio of 0.66 (95% *CI*: 0.49–0.90) and 0.61 (95% *CI*: 0.48–0.77) respectively. Conversely, in Model 2, there was no correlation between PA and the risk of DM in either the low or high RC groups. Moreover, there was no significant interaction between PA and RC in relation to DM risk (*p* > 0.05).

**Table 4:** Risk of DM by three groups of PA stratified according to two groups of RC*.

Two groups of RC	Three groups of PA	Model 1	*P*-value	Model 2	*P*-value
		*OR* (95% *CI*)		*OR* (95% *CI*)	
Low	Low	Reference		Reference	
	Moderate	1(0.75–1.34)	0.992	1.43(0.93–2.18)	0.010
	High	0.66(0.49–0.90)	0.009	0.841(0.52–1.33)	0.447
	*P*-trend	0.003		0.302	
High	Low	Reference		Reference	
	Moderate	0.86(0.67–1.10)	0.225	0.93(0.69–1.27)	0.656
	High	0.61(0.48–0.77)	< 0.001	0.70(0.49–1.01)	0.054
	*P*-trend	<0.001		0.052	
*P*_interaction_		0.733		0.270	

*All estimates were weighted. PA, physical activity; RC, remnant cholesterol; DM, diabetes mellitus; OR, odds ratio; CI, confidence interval; P _interaction_ is the value of interaction between PA and RC on DM. Molde 1: Did not adjust any covariates; Molde 2: Adjusted for age, PIR, FBG, BMI, DII, HOMA-IR, gender, race, marital status, education level, alcohol use; smoking status, hypertension, CVD, sleepiness level and health insurance.

### The mediating role of RC

**Figure 3:**
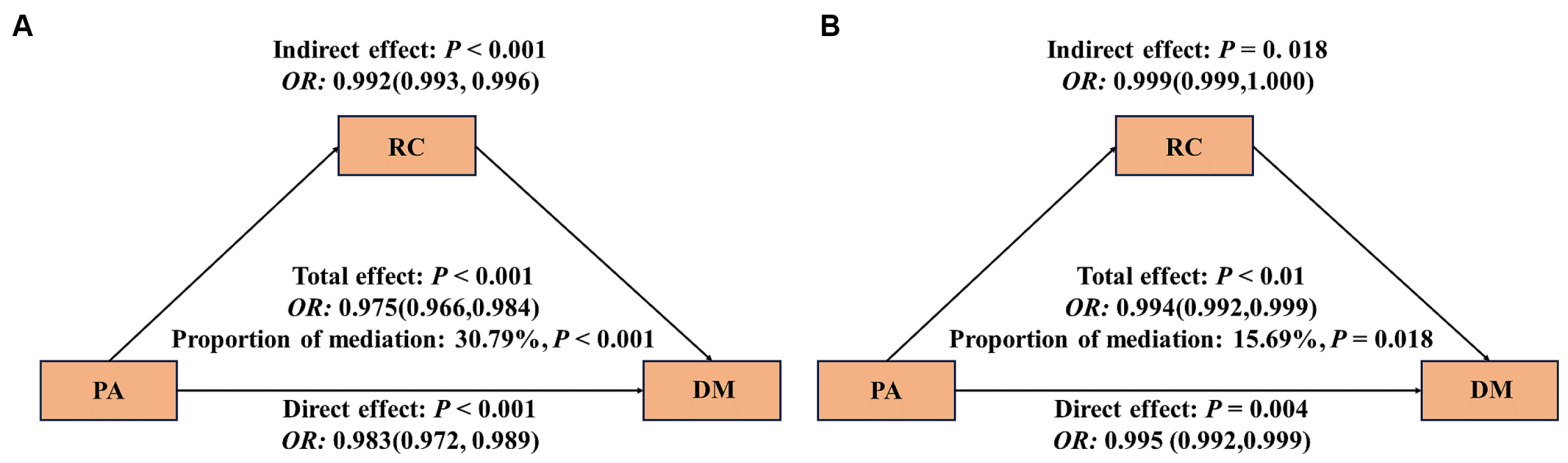
Mediating effect of RC between PA and DM. The 95% *CI* of these estimates was computed using the bootstrap method (1,000 samples); **(A)** did not adjust any covariates; **(B)** was adjusted for age, PIR, FBG, BMI, DII, HOMA-IR, gender, race, marital status, education level, alcohol use; smoking status, hypertension, CVD, sleepiness level and health insurance.

In Figure 3, it is evident that an increase in PA is linked to a reduction in the risk of DM. Approximately 30.79% of this effect can be attributed to a notable indirect impact associated with RC [*OR*: 0.992 (95%*CI*: 0.993, 0.996)] (as shown in Figure 3A). However, following the adjustment for covariates, the mediating effect of RC has weakened, accounting for 15.69%, and the indirect effect has statistical significance [*OR*: 0.999 (95%*CI*: 0.999, 1.000)] (Figure 3B). In addition, we will also include TG, HDL, LDL, and TC were used as mediating variables between PA and DM for mediation analysis. The results showed that after adjusting for covariates, TG had a mediating effect of 12.32%, HDL had a mediating effect of 8.92%, There is no mediating effect between LDL and TC (Supplementary Table S2).

## Discussion

In this extensive cross-sectional investigation into the influence of PA and RC on the likelihood of developing DM, we uncovered several crucial findings. Initially, we noticed a distinct inverse relationship between PA and the risk of DM. As PA levels increase, the likelihood of developing DM steadily diminishes. This pattern is especially evident in Model 3, which establishes a consistent dose–response connection between PA and the risk of DM. In addition, we found a positive correlation between high RC levels and DM risk, and observed a significant linear dose–response relationship. In models not adjusted for covariates, it appeared that RC played a substantial mediating role in the relationship between PA and DM. When we consider the covariate adjusted model, the mediating effect of RC on the relationship between PA and DM weakens. Finally, our analysis of interactions demonstrated the absence of a substantial interaction between PA and RC regarding the risk of DM development.

Our results show that there is a consistent negative correlation between higher levels of PA and the risk of DM. The observation results of the dose–response relationship between the three models support this point, indicating that the risk of DM decreases with the increase of PA. This finding is consistent with the existing literature, which emphasizes the protective role of regular PA in the prevention and management of DM. For instance, a study on the risk of DM among Kurdish populations in Iran identified PA as a protective factor, with higher daily PA levels associated with a reduced incidence of DM (27). Similarly, Brož et al. found that both aerobic and muscle-strengthening exercises were less frequent in the DM group in contrast to the non-diabetes group (28). In the Czech population, aged 25–64, a higher prevalence of DM was observed among individuals with low levels of PA. In addition, restricted cubic spline analysis reveals a nonlinear relationship between PA and DM risk, indicating the existence of a turning point beyond which the protective effect of PA becomes more significant. This nonlinear model emphasizes the importance of reaching a certain PA threshold to maximize the benefits of reducing the risk of DM.

In our study, compared with the non-diabetes group, the RC level of DM patients is higher, and there is a significant linear relationship between RC and DM. The higher RC level is associated with the increased risk of DM. Some studies also support the correlation between RC and DM from the side. A DM prevention cohort study (22) shows that the RC concentration in the pre diabetes group is significantly higher than that in the normal population. Additionally, in a single-center cohort study (29), it was found that elevated RC levels were independently linked to a higher risk of developing new DM (*HR*: 2.44, 95% *CI*: 1.50–3.89). It is worth noting that, Numerous research endeavors have corroborated the strong connection between PA and RC. In a controlled experiment with randomization (22), the experimental group witnessed a decline in non-high-density lipoprotein cholesterol levels, specifically RC, subsequent to engaging in resistance training. And the RC level of high-intensity occupational activities is lower than that of low-intensity non occupational activities (30). Another study also found that the RC levels of subjects who met the PA guidelines for PA intensity were lower than those who did not meet the PA guidelines (31). Overall, regular moderate intensity PA has been shown to regulate RC by preventing an increase in LDL cholesterol and triglyceride levels (32). Our findings also support this perspective, which will contribute to a better understanding of the intricate association between PA, RC, and DM. The mediation analysis indicates that approximately 30.79% of the protective effect of PA on DM risk can be attributed to a notable indirect impact associated with RC. This implies that the beneficial effects of PA on DM may, in part, operate through the modulation of residual cholesterol levels. Research has shown that RC directly or indirectly regulates insulin sensitivity by affecting lipid metabolism (33, 34), and high levels of PA have been shown to be associated with improving lipid metabolism and increasing insulin sensitivity in different populations (35–37), The increase in RC levels may interfere with normal lipid metabolism, leading to disruption of insulin signaling and thus increasing the risk of DM. The mediating role of RC may also involve the regulation of inflammation and oxidative stress (38, 39), and PA has been proven to have a positive impact on combating inflammation and reducing oxidative stress (40–42). The accumulation of RC may trigger inflammatory reactions and exacerbate oxidative stress, leading to the development of insulin resistance and DM.

It is worth noting that after adjusting the covariates, the mediating effect of RC decreased from the original 30.79–15.69%. It may be that the role of RC in the pathogenesis of DM is affected by other covariates. Some studies have found that BMI, smoking, PA, etc. play a mediating role between education level and type 2 diabetes (43). In a mediation analysis of DM neuropathy (44), HbA1c plays a strong mediating role. In addition, albumin, HDL-C, TG, apolipoprotein A, and C-reactive protein also play a mediating role. In our study, we also found that TG had a mediating effect of 12.32% and HDL had a mediating effect of 8.92%, but LDL and TC did not show a mediating effect. Further research is needed to explain these interactions. In addition, the interaction between PA and RC on DM risk is not significant (Table 4), and in stratified analysis, regardless of RC stratification, the effect between PA and DM risk is consistent. This means that our results are robust.

This study has several advantages. It utilizes a representative and extensive sample of data, and it examines the association between PA and DM using multiple analytical approaches. Additionally, it investigates the potential role of RC in this association. Our study reveals a distinct and independent correlation between PA and the risk of DM, Importantly, there is no interaction between RC and PA when it comes to the risk of DM. In other words, RC does not influence the relationship between PA and DM. This finding has significant implications for clinical practice and public health. By increasing PA, a modifiable factor, we can effectively prevent or alleviate symptoms of DM. Nevertheless, it is crucial to acknowledge the limitations of our research. Firstly, our study measures PA as a cumulative sum of all physical activities in daily life. It does not distinguish between different types of PA, such as vigorous work activity, recreational activities, or moderate work and recreational activities, including walking or cycling for transportation (45). Consequently, we are unable to determine the specific impact of each type of PA on DM. Secondly, the results of our study are derived from American adults and may not accurately reflect the true situation of other populations. Additionally, as a cross-sectional study, our research cannot establish a causal relationship between PA and DM. It is imperative to conduct further prospective on-site intervention studies to provide stronger evidence on the association between PA and DM.

Overall, this study provides evidence supporting the protective role of PA in reducing DM risk, while emphasizing the potential mediating role of RC. Understanding the complex relationship among PA, RC and DM risks is crucial for developing targeted interventions and personalized DM prevention and management methods.

## Data availability statement

The original contributions presented in the study are included in the article/Supplementary material, further inquiries can be directed to the corresponding authors.

## Ethics statement

Ethical approval was not required for the study involving humans in accordance with the local legislation and institutional requirements. Written informed consent to participate in this study was not required from the participants or the participants' legal guardians/next of kin in accordance with the national legislation and the institutional requirements.

## Author contributions

ZY: Data curation, Formal analysis, Writing – original draft. HC: Data curation, Formal analysis, Writing – original draft. FL: Methodology, Visualization, Writing – review & editing. JZ: Investigation, Writing – review & editing. ShiW: Formal analysis, Methodology, Writing – review & editing. ShuW: Formal analysis, Investigation, Visualization, Writing – review & editing. YC: Formal analysis, Methodology, Validation, Writing – review & editing. ZM: Data curation, Resources, Writing – review & editing. LL: Investigation, Validation, Writing – review & editing. DK: Supervision, Writing – review & editing. YD: Resources, Writing – review & editing.

## Footnotes

^1^https://wwwn.cdc.gov/Nchs/Nhanes

^2^http://www.R-project.org
