# Supplementary material for: Role of remnant cholesterol in the relationship between physical activity and diabetes mellitus: an intermediary analysis
Source: Front Public Health. 2024 Mar 12;12:1322244. doi: 10.3389/fpubh.2024.1322244 (PMC10963391; doi:10.3389/fpubh.2024.1322244)
Supplement: Supplementary file 1 [file Table_1.docx]

**Supplementary Table 1** DM risk based on RC grouping*

| Characteristics | Model 1  *OR* (95% *CI*) | Model 2  *OR* (95% *CI*) | Model 3  *OR* (95% *CI*) |
| --- | --- | --- | --- |
| **Two groups of RC** |  |  |  |
| Low | Reference |  |  |
| High | 2.48(2.15-2.87) | 2.34(1.99-2.75) | 1.29(1.02-1.62) |
| *P*-trend | < 0.001 | < 0.001 | 0.03 |
| **For 1-SD increase** | 1.69(1.58-1.82) | 1.69(1.55-1.86) | 1.15(1.01-1.31) |

* All estimates were weighted;

Abbreviations: RC, remnant cholesterol; DM, diabetes mellitus; *OR*, odds ratio; *CI*, confidence interval;

Model 1: Did not adjust any covariates; Model 2: Adjusted for age, gender, race, marital status, education level; Model 3: Adjusted for age, PIR, FBG, BMI, DII, HOMA-IR, gender, race, marital status, education level, alcohol use; smoking status, hypertension, CVD, sleepiness level and health insurance, and PA; For 1-SD increase: Using RC with natural logarithms.

**Supplementary Table 2** The mediating role of each variable between PA and DM*

| Mediating variables | Prop Mediated | Effect | *OR* (95% *CI*) | *P* |
| --- | --- | --- | --- | --- |
| TG | 12.32%  *P* = 0.018 | Total effect | 0.994(0.992-0.999) | 0.01 |
|  |  | Indirect effect | 0.999(0.999-1.000) | 0.008 |
|  |  | Direct effect | 0.995(0.993-0.999) | 0.012 |
| HDL | 8.92%  *P* = 0.016 | Total effect | 0.993(0.992-0.999) | 0.014 |
|  |  | Indirect effect | 0.999(0.999-1.000) | 0.002 |
|  |  | Direct effect | 0.994(0.992-0.999) | 0.02 |
| LDL | 0.86%  *P* = 0.606 | Total effect | 0.993(0.991-0.999) | 0.008 |
|  |  | Indirect effect | 1.000(0.999-1.000) | 0.602 |
|  |  | Direct effect | 0.993(0.991-0.999) | 0.008 |
| TC | 5.05%  *P* = 0.184 | Total effect | 0.993(0.991-0.999) | 0.002 |
|  |  | Indirect effect | 1.000(0.999-1.000) | 0.182 |
|  |  | Direct effect | 0.992(0.991-0.999) | 0.006 |

* All estimates were weighted;

The 95% *CI* of these estimates was computed using the bootstrap method (1,000 samples); Mediation analysis adjusted for age, PIR, FBG, BMI, DII, HOMA-IR, gender, race, marital status, education level, alcohol use; smoking status, hypertension, CVD, sleepiness level and health insurance.
