# Supplementary material for: Role of remnant cholesterol in the relationship between physical activity and diabetes mellitus: an intermediary analysis
Source: Front Public Health. 2024 Mar 12;12:1322244. doi: 10.3389/fpubh.2024.1322244 (PMC10963391; doi:10.3389/fpubh.2024.1322244)
Supplement: Supplementary file 2 [file Image_1.pdf]

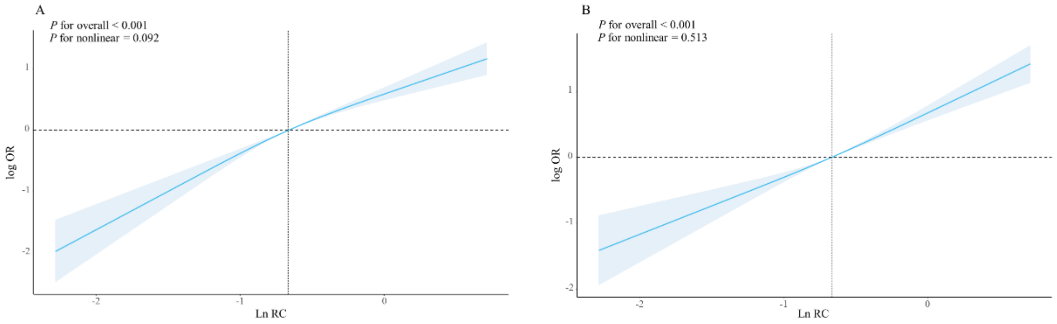

Supplementary Figure 1: The dose–response relationships of RC with DM in all participants. Results were from restricted cubic spline models; A The dose-response relationship between RC and DM without adjusting for any covariates; B the dose-response relationship between RC and DM adjusted for age, gender, race, marital status, education level.
